# Inequalities in accelerated cognitive decline: Resolving observational window bias using nested non‐linear regression

**DOI:** 10.1002/alz.14053

**Published:** 2024-07-12

**Authors:** Sean A. P. Clouston, Douglas W. Hanes, Dylan M. Smith, Lauren L. Richmond, Marcus Richards, Bruce Link

**Affiliations:** ^1^ Program in Public Health, Renaissance School of Medicine Stony Brook University Stony Brook New York USA; ^2^ Department of Family, Population, and Preventive Medicine, Renaissance School of Medicine Stony Brook University Stony Brook New York USA; ^3^ Department of Psychology Stony Brook University Stony Brook New York USA; ^4^ MRC Unit for Lifelong Health and Ageing at UCL University College London London UK; ^5^ Department of Sociology University of California at Riverside Riverside California USA; ^6^ Department of Public Policy University of California at Riverside Riverside California USA

**Keywords:** Alzheimer's and related diseases, cognitive pathology, longitudinal cognitive decline, parametric segmented modeling

## Abstract

**INTRODUCTION:**

Limited observational windows lead to conflicting results in studies examining educational differences in Alzheimer's disease and related dementias (ADRD) risk, due to observational window bias relative to onset of accelerated cognitive decline. This study tested a novel model to address observational window bias and tested for the presence and sources of disparities in accelerated cognitive declines due to ADRD.

**METHODS:**

The sample examined 167,314 cognitive assessments from 32,441 Health and Retirement Study participants. We implemented a parametric non‐linear nested longitudinal regression and reported multivariable‐adjusted nodal incidence ratios (aNIR).

**RESULTS:**

University degrees were associated with lower incidence (aNIR = 0.253, 95% confidence interval [CI] = [0.221 to 0.289], *p* < 0.001), while black participants had a higher incidence (aNIR = 1.995, [1.858 to 2.141], *p* < 0.001) of accelerated cognitive decline, adjusting for demographic, sociobehavioral, and medical risk factors. Sex‐stratified analyses identified diminished educational returns for women and increased incidence among minoritized women.

**DISCUSSION:**

Addressing observational window bias reveals large social inequalities in the onset of accelerated cognitive declines indicative of ADRD.

**Highlights:**

- This study identifies observational window bias as a source of conflicting results among previous studies of educational achievement in Alzheimer's disease and related dementias (ADRD) disparities.
- The study locates preclinical accelerated cognitive decline, which is indicative of ADRD while occurring 10+ years prior to symptom onset, as a site to study ADRD disparities that mitigates observational window bias.
- A novel method, nested non‐linear regression, is developed to test for differences in the onset of accelerated cognitive decline.
- Educational and racial/ethnic disparities are demonstrated in the onset of accelerated cognitive decline, as are their intersecting differences with sex/gender.

## 1 BACKGROUND

Alzheimer's disease and related dementias (ADRD) impacted 6.7 million Americans in 2023^1^ and were listed as the underlying cause of 119,399 deaths in 2021.^2^ ADRD is unequally burdensome, affecting populations with lower socioeconomic status (SES) and racial/ethnic minorities at higher rates than others. Prior work argued that inequalities reflect lifelong differences in cognitive performance emerging from childhood,^3^ partly because underinvestment in impoverished communities lowers educational quality.^4^, ^5^ Disparities may also emerge from stressful exposures and increased ADRD risk factors, including cardiovascular disease (CVD), alcohol consumption, and smoking.^6^

Most ADRD risk factors are correlated with educational attainment, so we expect a robust correlation between education and ADRD. Yet, studies of the education–ADRD association show mixed results.^7^ If educational attainment is associated with later‐life cognition outcomes, there are several likely reasons: Education directly increases lifetime cognitive performance,^7^ as shown in a recent Mendelian randomization study.^8^ Indirectly, markers of socioeconomic status (SES), including educational attainment, also predict the distribution of ADRD risk factors, that is, the “risk of risks.”^5^, ^9^, ^10^, ^11^, ^12^ Education is linked to the aforementioned factors,^6^ as well as increased earnings and wealth, occupational advancement, intellectually stimulating work, improved working conditions, and geographic mobility,^13^, ^14^, ^15^ and education enables cognitively protective behaviors.^16^ Thus, an association between education and ADRD is to be expected.

Yet, both meta‐analyses^16^ and coordinated analyses of multiple samples^17^ have reported similar rates of cognitive decline in more‐ and less‐educated individuals, results inconsistent with the education–ADRD associations posited earlier. Studies that used the same methods to study inequalities by sex/gender^18^ and race/ethnicity^19^ also failed to show different rates of decline by education. In response, some have hypothesized that these contradictory findings are due to preserved differences in cognitive performance into old age; others suggested that educational and related disparities in underdiagnosis rates biased diagnostic studies.^20^

Reliance on diagnosable or clinical cognitive or functional symptoms is likely central to the contradictory findings regarding education and ADRD. ADRD are usually diagnosed when cognitive decline is severe enough to impede the ability to function in daily life,^21^ often proxied in studies by an ADRD or mild cognitive impairment (MCI) cutoff on assessment scores.^22^ Many studies will then examine the time until their onset.^23^

**FIGURE 1:**
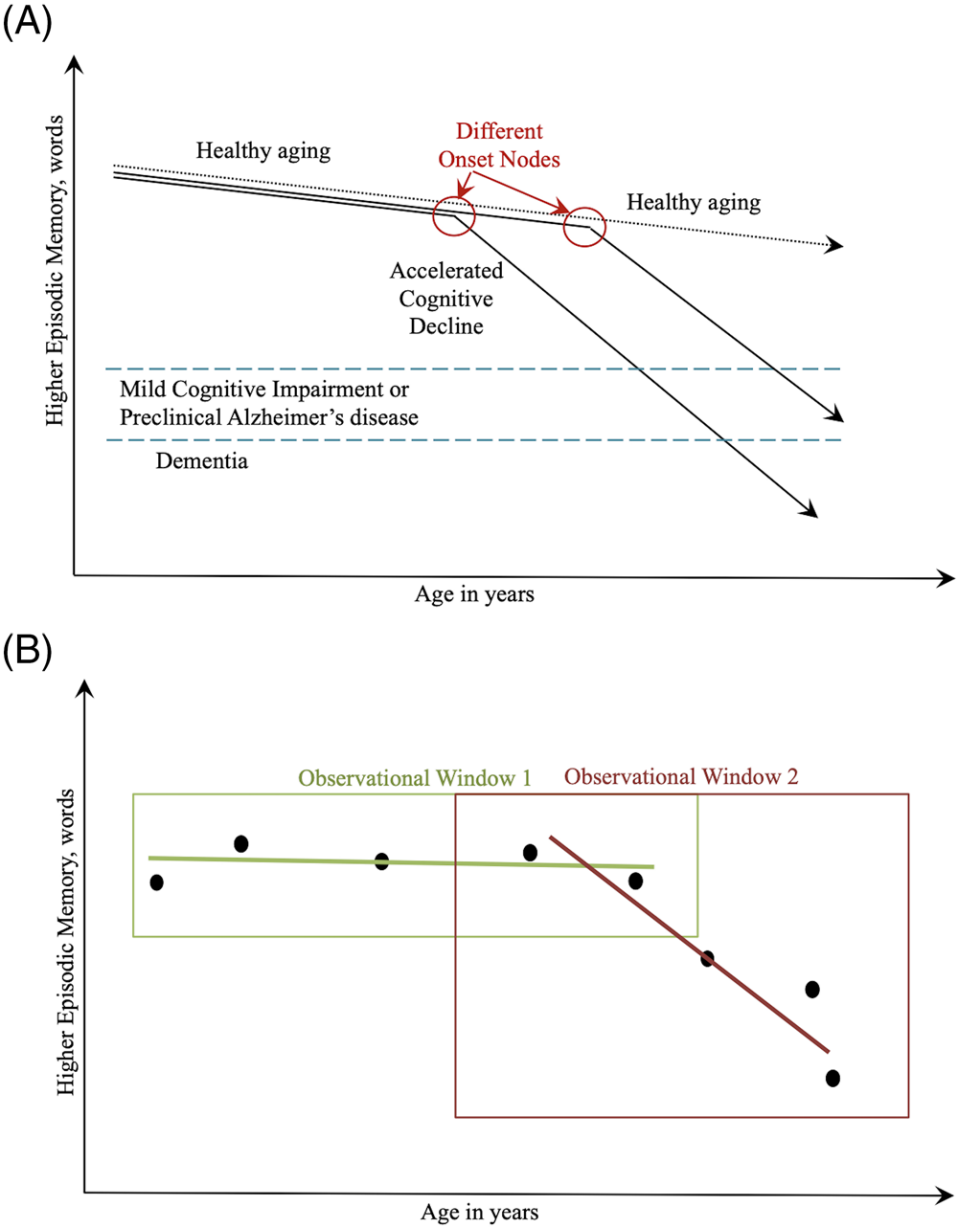
Theoretical figure expanding on the problem of accelerated cognitive declines in observational longitudinal data. Panel A shows what ADRD researchers expect from cognitive declines in the time leading up to MCI and ADRD diagnosis. Panel B shows what data are observed to look like in longitudinal data but also highlights the problem with randomly occurring observational windows that lead to observational window bias. ADRD, Alzheimer's disease and related dementias; MCI, mild cognitive impairment.

One potential problem in existing studies of cognitive aging, then, is the *observational window* they use. Different studies capture different windows of observation (Figure 1B), increasing noise and bias. Specifically, the observed rate of cognitive decline could be drastically different depending solely on an unobserved third variable: when during their decline trajectory a participant was recruited into the study. Someone might have either a normal (window 1) or accelerated (window 2) observed rate, depending on which period their observational window captures. Resolving this so‐called observational window bias is critical: longitudinal models predominantly focus on linear random slopes (equivalent to the red and green trend lines in Figure 1B). In terms of bias, those same differences hypothesized to lead to earlier ADRD onset are also associated with premature mortality^24^; the mixed results of existing studies may also be due, then, to undetected survivor effects.

One promising avenue is to expand the window of analysis before diagnosable ADRD or even MCI. In neuropathological studies, more education is consistently associated with decreased levels of amyloidosis in preclinical studies,^25^ alongside reduced risk of secondary tauopathy^8^ but more severe outcomes in the presence of amyloidosis.^26^ Prediagnostic or preclinical ADRD is differentiable from normal cognitive aging by the onset of accelerated cognitive decline, ^27^ resulting in ADRD after a 10‐ to 20‐year latency period^28^ (Figure 1A). The onset and rate of accelerated cognitive decline, definitive of ADRD, present additional sites of analysis to detect ADRD disparities that address potential sources of noise and bias arising from reliance on clinical onset. Using a pattern‐recognition protocol, one study found higher education was associated with delayed onset of patterns of accelerated cognitive decline.^29^

## 2 METHODS

The goal of this study was to test the potential to resolve the previously described conflicts in existing ADRD disparities research by addressing observational window bias by extending the window to include accelerated cognitive decline. We developed a novel analytic method, nested non‐linear regression, and tested to determine whether it could better measure the relationship between education and cognitive disparities and, if so, whether demographic factors, CVD, or lifestyle behaviors helped to explain this association. We used a large, longitudinal, nationally representative sample of older Americans, the Health and Retirement Study (HRS; 1998 to 2020).^30^ This sample is useful because the windows of observation are broad, starting at age 51 years, thereby coming closer to capturing the start of accelerated decline within the observational window rather than only segments of it as depicted in Figure 1B. Specifically, we examined predictors of the onset of accelerated cognitive declines using a layered survival modeling technique. We hypothesized that (a) lower education and minoritized race/ethnicity would be associated with a more rapid onset of accelerated cognitive declines indicative of the onset of ADRD and (b) that the association between these markers of inequality would be partially explained by the unequal distribution of distress, CVD, and health behaviors. In addition, since gender has been shown to modify both the potential benefits of education and the impact of minority status, we replicated models stratified by sex/gender.

**RESEARCH IN CONTEXT**

1. **Systematic review**: A literature review using PubMed revealed that several robust measures of social inequality, including education, socioeconomic status (SES), and race/ethnicity, were consistently associated with nearly all known risk factors for ADRD but not with rates of cognitive decline. Reviews presenting such conflicting results were cited.
2. **Interpretation**: Our findings imply that methods that are attuned to within‐person differences in the timing of onset of cognitive declines consistent with Alzheimer's disease could reveal strong social inequalities that were partially mediated by known risk factors for ADRD.
3. **Future directions**: More studies are needed to understand the importance of observational window bias on longitudinal studies. Further studies are warranted examining risk factors for accelerated declines in cognition as a proxy for the onset of ADRD.

### 2.1 Sample

We analyzed 12 waves of data from the HRS (1998 to 2020; initial response rate = 81.6%).^31^ The HRS is a biannual, nationally representative study that has collected cognitive functioning consistently, using the same measures since 1998 (wave 3). Participants become age‐eligible at age 51; starting in 1998, the study became steady‐state, recruiting subsequent cohorts of age‐eligible participants every third wave (ie, every 6 years).

### 2.2 Inclusion/exclusion criteria

We focused on eligible participants aged 51 years and older with at least two valid waves of the 10‐word episodic memory task was implemented in wave 3. We excluded participants with missing demographic data. To ensure that we captured a pre‐rapid‐decline period, we also excluded individuals whose mental status (numeracy and orientation; max score 10) assessment, when available, suggested that they already had evidence of cognitive functional limitations (mental status score <6) and anyone who showed rapid cognitive decline, defined as a more than four‐word loss between their first and second assessments.

### 2.3 Outcome

*Episodic memory* is a core feature of cognitive decline relating to ADRD.^32^ To measure episodic memory in the HRS, participants were verbally asked to learn a list of 10 semantically unrelated words and immediately repeat them to the interviewer upon completion (immediate free recall). After intermediate distraction questions, including a verbal fluency task, the participants were asked to correctly recall the entire list of words (delayed recall). Episodic memory is the summation of both immediate and delayed recall scores (20 points max).

### 2.4 Domains of social inequality

*Educational attainment* was measured at the individual level as the highest degree achieved. We categorized educational attainment into four groups: less than high school, high school diploma or equivalent, some college but no college degree, or at least a college degree. *Race/ethnicity* was measured using two self‐reported questions (white, Black, or other race; and Hispanic ethnicity) that we combined into four mutually exclusive groups: white, Black, Hispanic, and Other. *Male* sex/gender was also measured by self‐report at the study's commencement; women were made the reference group because males have lower verbal episodic memory than females^33^ and worse overall uptake of heart‐healthy behaviors,^34^ but females often have a higher burden of cerebral tauopathy and may have more rapid consequent cognitive aging.

### 2.5 Sociobehavioral factors

*Household wealth* was measured as an indicator of access to shared resources in old age. It includes information about money from all sources at the household level including employment, savings, home(s), individual retirement accounts, pensions, social security, and so forth, less all debts. Wealth was highly skewed, so we used the logarithmically transformed version (ln(wealth in USD+1)). *Working status* is often used as a proxy for the healthy worker effect, in part because those who retire often do so for reasons related to worsening health.^35^ *Height* (meters) has been a reliable proxy for stable access to food in childhood that can have lifelong consequences for aging.^36^ *Smoking status* was a combination of smoking history and current smoking status. We stratified smoking status into those who never smoked, those who formerly smoked, and those who currently smoke. *Alcohol intake* was included in this study as the average number of alcoholic drinks that a participant reported over the past week.

### 2.6 Medical factors

A history of *stroke* and the *CVD burden* are strong correlates of a clinical diagnosis of ADRD. *Cardiovascular disease burden* is a summation including a history of hypertension, heart attack, and diabetes. Since many studies report potential associations between lifetime depressive symptoms and earlier onset of ADRD, we included depressive symptoms as measured using an eight‐item version of the Centers for Epidemiologic Studies in Depression scale,^37^ available in the HRS and its sister studies.^38^ However, since there is a concern that mild depressive symptoms can be worsened by the presence of neurodegenerative disease, we only included depressive symptoms at each participant's baseline visit.

### 2.7 Time scale

*Unfamiliarity* with neurocognitive tests and HRS testing circumstances is believed to result in worse scores on the first testing occasion,^39^ so a dichotomous flag variable was included in all models to identify every individual's first testing occasion. *Time in study*: Surveys at HRS occur, on average, every 2 years, but visits occur sporadically in that period, so we relied on the year and month of the interview to measure time in all models. Before acceleration was modeled, a linear time trend was assumed. *Age* in years, centered at age 50, was included to consider the normal rate of cognitive change absent or prior to accelerated decline.

### 2.8 Statistical analysis

We began by providing sample descriptive characteristics for the whole sample and stratified by educational attainment. To provide context in terms of missing data and attrition, we provided sample characteristics among those who did not complete the most recent wave (ie, attritors) and compared those who completed some, but not all, data collection efforts to those who completed all data collection. Next, since many researchers use a variety of models, we interrogated model fit while comparing several models with different modeling structures. Notably, we conducted analyses using an autoregressive structure compared to random intercepts and further examined the utility of random slopes using time in the study and age as the time scale. We reported model fit using Akaike's information criterion (AIC) and the Bayesian information criterion (BIC) and calculated likelihood weights based on Akaike's information criteria as a proxy for the probability that a model improved over prior attempts, and these are reported as W‐values.^40^ We also estimated ∼*R* ^2^ and the estimated likelihood ratio (LR), and accompanying *p* value, using the chi‐squared statistic to compare between models.

Next, we fit the nested cognitive aging and survival model following Equation 1:

(1) $$C_{it}=X_{0}\beta_{0}\;+X_{t}\vartheta_{1t}+\varphi *{\left(A_{it}-\left(X\delta_{i}-\theta A_{0}\right)\right)}^{+}+\gamma_{0i}+\gamma_{1i}t+\varepsilon_{it}$$

where C refers to the cognitive outcome, which varies between individuals (*i*) and changes over time (*t*). *A* refers to age, and ***β*** references an array of regression coefficients absorbing important time‐invariant covariates, while* θ_it_* captures time‐varying confounders, and φ is the rate of accelerated cognitive decline after the node. Age‐related changes occurring throughout the accelerated segment of the model were captured using the term ${\left(A_{it}-\left(X\delta_{i}-\theta A_{0}\right)\right)}^{+}$, where for consistency with others in nested regression modeling regions we have defined the function ${\left(x\right)}^{+}\;:\;=\;\max\left(x,0\right)$. We included g_0i_ to account for individual differences in lifetime cognitive function encoded as the intercept. We also included g_0i_t to identify individual random slopes and incorporated an adjustment for the covariation between random intercepts and slopes to account for regression to the mean. We fit this model using mixed‐effects non‐linear multilevel regression using quasi‐code shown in Supplemental Quasi‐Code 1.

We fit the model first using a demographically adjusted model integrating adjustments for age on the rate of change in cognition. We considered the impact of unfamiliarity on baseline, and we additionally adjusted for the difference in incidence due to sex/gender. Second, a sociobehavioral and medical risk‐factor model was then fit to account for differences between individuals in the importance of various risk factors including household wealth (ln‐$+1) at baseline, CVD burden, stroke, whether the person worked at baseline, the level of depressive symptoms at baseline, height, smoking status, and alcohol intake. Here, we included both a history of stroke and CVD burden at baseline and as time‐varying incident conditions. From the survival model we report exponentiated coefficients referring to the variable placement of nodes indicating the start of the accelerated model and refer to these as multivariable‐adjusted nodal incidence ratios (aNIRs) and report 95% confidence intervals. Since previous studies reported worse secondary outcomes but better lifetime cognition in women with AD, we included a sex‐stratified analysis. In addition, since previous research found stronger educational benefits for women as well as worse overall impact of minoritized status, we compared the size of the coefficients for men and women using coefficient comparison ratios (CCRs) with 95% confidence intervals.

All models were run using Stata 17/MP [StataCorp] using the menl package. Population weights were provided by the HRS to account for sampling structure.

## 3 RESULTS

After applying inclusion/exclusion criteria, we retained data from 32,441 participants (97.3% of eligible individuals), who provided data on 167,314 testing occasions for an average of 5.16 observations per participant for analysis (Figure S1).

Analyses of the rate of attrition (Table S1) revealed that working status, higher cognition, increased levels of wealth, and alcohol intake at baseline were associated with a lower risk of attrition, while older age, male sex/gender, higher depressive symptoms, increased CVD burden, and a history of stroke were associated with increased hazards of attrition. Analysis of sample inclusion criteria (Table S2) indicated that those who provided complete data were more highly educated, had higher cognition, were less likely to report Hispanic race/ethnicity, had lower levels of wealth, and higher depressive symptoms.

We began by describing the sample characteristics and important covariables (Table 1). The average participant was 60 years old at baseline, measured 1.69 m (5 ft 6 in.) tall and was working at the first measurement occasion. Most participants were non‐Hispanic white (74.3%), and approximately half were men (50.48%). Stratified comparisons across educational categories were all statistically significant, as described in the table note (*p* < 1E‐06), and showed large gradients in episodic memory across groups. Results suggest that those with higher education are younger, more likely to be non‐Hispanic white, had higher wealth, were more likely to be working, were taller, had lower CVD burden, and were less likely to report a history of stroke or any symptoms of depression at baseline, and many fewer reported currently smoking.

**TABLE 1:** Sample characteristics for health and retirement study.

	Whole sample (*N* = 32,441)	Less than high school (*n* = 7257)	High school diploma (*n* = 10,828)	Some college (*n* = 7745)	University degree (*n* = 6611)
Characteristic	Mean (SD)/*N* (%)	Mean (SD)/*N* (%)	Mean (SD)/*N* (%)	Mean (SD)/*N* (%)	Mean (SD)/*N* (%)
Episodic memory, words	10.3 (3.33)	8.03 (3.23)	10.06 (3.12)	10.71 (3.04)	11.78 (3.02)
First cognitive assessment	22,754 (70.14%)	3784 (52.14%)	7176 (66.27%)	5997 (77.42%)	5296 (80.11%)
Age, years	59.63 (9.32)	64.65 (11.27)	59.87 (9.13)	58.08 (8.22)	57.44 (7.61)
Race/ethnicity					
White	24,199 (74.59%)	4071 (56.1%)	8467 (78.19%)	5863 (75.7%)	5403 (81.72%)
Black	3764 (11.6%)	1281 (17.65%)	1207 (11.15%)	959 (12.38%)	475 (7.18%)
Other	1605 (4.95%)	277 (3.82%)	404 (3.73%)	394 (5.09%)	472 (7.14%)
Hispanic	2874 (8.86%)	1628 (22.43%)	750 (6.93%)	529 (6.84%)	262 (3.96%)
Male	16,281 (50.19%)	3523 (48.54%)	5133 (47.4%)	3846 (49.66%)	3663 (55.41%)
Wealth, ln(USD)	7.95 (2.97)	5.93 (3.37)	7.69 (2.79)	8.18 (2.7)	9.46 (2.17)
Height, m	1.7 (0.1)	1.68 (0.1)	1.7 (0.1)	1.71 (0.1)	1.72 (0.1)
Cardiovascular disease	0.74 (0.82)	0.97 (0.9)	0.76 (0.83)	0.72 (0.81)	0.57 (0.74)
Stroke	1429 (4.4%)	590 (8.13%)	470 (4.34%)	312 (4.02%)	151 (2.28%)
In labor force	20,580 (63.44%)	2707 (37.3%)	6443 (59.5%)	5393 (69.63%)	5306 (80.26%)
Baseline depressive symptoms	1.48 (1.99)	2.12 (2.24)	1.56 (2.03)	1.39 (1.93)	1.01 (1.66)
Smoking status					
Never smoker	13,838 (43.03%)	2578 (35.87%)	4189 (39.06%)	3128 (40.68%)	3638 (55.44%)
Former smoker	11,757 (36.56%)	2684 (37.35%)	3976 (37.07%)	2839 (36.93%)	2296 (34.99%)
Current smoker	6564 (20.41%)	1924 (26.78%)	2561 (23.88%)	1722 (22.39%)	628 (9.57%)
Alcohol intake, drinks/week	1.28 (2.04)	0.77 (1.74)	1.17 (2.01)	1.34 (2.03)	1.71 (2.2)

*Note*: Non‐parametric trend tests confirmed that all variables shown were statistically significantly different between educational categories (*p* < 1E‐06). Descriptive characteristics are weighted to US population.

Before proceeding, we interrogated model fit by comparing several models with different modeling structures in Table 2 (results from the demographics‐only models reported in Table S3). We compared results to several modeling structures but especially focused on comparisons with the most complex model, which used linear and quadratic acceleration slopes. Ultimately, these results validated work suggesting that the nested non‐linear regression was the best‐fitting and most efficient model.

**TABLE 2:** Model fit statistics using different modeling assumptions comparing consecutive models to the prior model.

Measure	Null model	Quadratic age model with random slopes (wave) and all covariates	Segmented survival model with random slopes (wave) and all covariates
Akaike information criterion	892,233.4	818,558.5	797,708.4
Bayesian information criterion	892,243.5	818,870.1	797,909.4
W‐value	<0.001	<0.001	<0.001
Likelihood ratio	25,451.8	73,734.9	20,828.1
∼*R* ^2^	0.028	0.108	0.131
*p* value	<0.001	<0.001	<0.001
Number of predictors in the model	1	31	20

**FIGURE 2:**
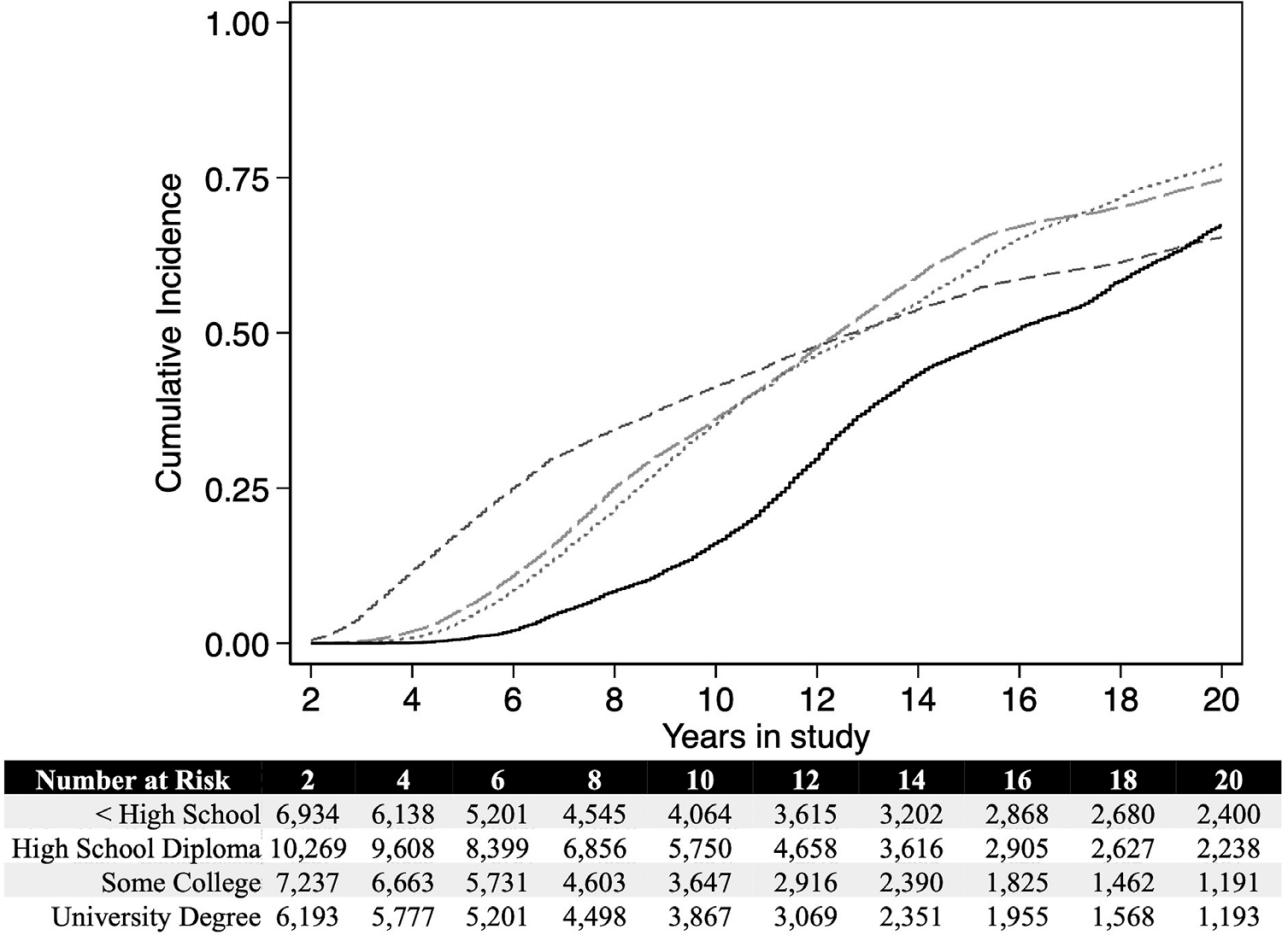
Cumulative incidence and risk table showing time until onset of accelerated cognitive declines indicative of Alzheimer's disease or a related dementia. Incidence curves are stratified by educational attainment into four groups to show participants with <high school diploma (dashed gray line), high school diploma (long dashed gray line), some college (gray dotted line), and university degree (solid black line).

In Figure 2, we show the main educational association. Here, all results are statistically significant and are presented in Table 3. Our results indicate that there are sizable differences in the onset of accelerated cognitive declines by educational attainment but also suggest that these differences even out as individuals get older and spend longer in the study. While not evident on this graph, the median time until the onset of accelerated cognitive decline was 10.25 years.

**TABLE 3:** Results from nested non‐linear regression showing multivariable‐adjusted nodal rates and corresponding confidence intervals and *p* values, Health and Retirement Study.

	Demographic model			Sociobehavioral and medical model		
Measure	aNIR	95% CI	*p*	aNIR	95% CI	*p*
Educational attainment						
Less than high school	1.000			1.000		
High school diploma	0.172	0.127 to 0.233	<0.001	0.374	0.328 to 0.426	<0.001
Some college	0.130	0.096 to 0.178	<0.001	0.310	0.271 to 0.354	<0.001
University degree	0.097	0.071 to 0.133	<0.001	0.253	0.221 to 0.289	<0.001
Race/ethnicity						
White	1.000			1.000		
Black	3.099	2.798 to 3.432	<0.001	1.995	1.858 to 2.141	<0.001
Other	1.903	1.665 to 2.174	<0.001	1.566	1.395 to 1.757	<0.001
Hispanic	2.154	1.936 to 2.397	<0.001	1.672	1.524 to 1.835	<0.001
Male	1.667	1.61 to 1.726	<0.001	1.834	1.754 to 1.917	<0.001

*Note*: Results from nested non‐linear regression models. The models also adjust for random intercepts and random slopes. An unstructured covariance matrix was included to model the potential regression to the mean. We also account for variance in the intercepts attributable to the range of confounders shown but also including estimates of test unfamiliarity. Stroke and CVD were both included as time‐varying predictors. Model 1 additionally adjusts for sex/gender and age. All models additionally adjust for wealth (ln‐$) at baseline, CVD burden, stroke, whether the person worked at baseline, level of depressive symptoms at baseline, height, smoking status, and alcohol intake. Analyses are weighted to US population.

Table 3 presents results from the nested non‐linear regression analyses: The first model only includes results for educational attainment, race/ethnicity, and sex/gender; the second also included the sociobehavioral and medical risk factors described earlier. Overall, analyses revealed that when controlling for potential confounders, both race/ethnicity and educational attainment remained statistically significant predictors of the onset of ADRD. While not shown in Table 3, additional risk factors included shorter height, being outside the labor force at baseline, higher depressive symptoms, and smoking status. However, our results also suggested that adjusting these factors reduced the association between both education and race/ethnicity on cognitive outcomes. For example, the association between a university degree compared to having less than a high school degree and the onset of accelerated cognitive decline was reduced by 19.5%. The racial/ethnic disparity between Black and white participants was reduced by approximately 52.4%, while the white–Hispanic disparity was only 36.7% explained by covariates.

We replicated results stratified by sex/gender and reported risk ratios to examine whether associations found in male participants are similar in effect to those reported in female participants (Table 4). Overall, we found that the importance of educational attainment to risk of onset was similar in females and males but that the benefit from higher education was less for females (CCR = 1.365, *p* < 0.001). Similarly, we found that the CCR was higher in women compared to men across all racialized groups, indicating that the risk associated with minoritized identities was stronger in females.

**TABLE 4:** Results from nested non‐linear regression showing multivariable‐adjusted nodal incidence ratios and corresponding confidence intervals and *p*‐values, stratified by gender, for participants in the health and retirement study.

	Sociobehavioral and medical model in males			Sociobehavioral and medical model in females			Male–female comparison	
Measure	aNIR	95% CI	*p*	aNIR	95% CI	*p*	CRR	*p*
Educational attainment								
Less than high school	1.000			1.000				
High school diploma	0.403	0.331 to 0.490	<0.001	0.438	0.384 to 0.499	<0.001	1.088	0.304
Some college	0.318	0.259 to 0.392	<0.001	0.373	0.326 to 0.426	<0.001	1.171	0.067
University degree	0.236	0.190 to 0.294	<0.001	0.323	0.281 to 0.370	<0.001	1.365	<0.001
Race/ethnicity								
White	1.000			1.000				
Black	1.698	1.517 to 1.899	<0.001	2.162	1.972 to 2.371	<0.001	1.274	<0.001
Other	1.304	1.114 to 1.527	0.001	1.752	1.486 to 2.065	<0.001	1.343	<0.001
Hispanic	1.386	1.220 to 1.575	<0.001	1.832	1.620 to 2.072	<0.001	1.322	<0.001

*Note*: Results from sociobehavioral and medical model (Table 3), stratified by sex/gender. The models also adjust for random intercepts and random slopes. An unstructured covariance matrix was included to model the potential regression to the mean. We also account for variance in the intercepts attributable to the range of confounders shown, but also including estimates of test unfamiliarity. Stroke and CVD were both included as time‐varying predictors. All models adjust for sex/gender, age wealth (ln‐$) at baseline, CVD burden, stroke, whether person worked at baseline, level of depressive symptoms at baseline, height in meters, smoking status, and alcohol intake. Analyses are weighted to US population.

## 4 DISCUSSION

Longitudinal studies of cognitive decline are highly sensitive to within‐person acceleration in the rate of aging because observational windows are not sensitive to the location of this aging. Using a novel analytic approach that corrects for observational window bias to identify accelerated cognitive decline consistent with ADRD, we observed strong associations between the onset of accelerated cognitive decline and both educational attainment and race/ethnicity, enabling us to resolve the conflicting findings in earlier studies regarding the education–ADRD association. Known risk factors for ADRD partially explained these associations. We also confirmed that these analyses improved model fit over other linear and quadratic models.

This study leveraged previous results from proof‐of‐concept studies to specify a non‐linear nested regression, which incorporated survival modeling, to account for multiple covariates including medical and behavioral risk factors.^29^, ^41^, ^42^, ^43^ We previously found that studies of cognitive decline must consider the importance of accelerated cognitive decline in longitudinal studies of participants, as opposed to methods that assume linear declines or diagnose ADRD at a set threshold. This study also replicated our previous results demonstrating that our method improved model fit over the quadratic. Our findings here support those earlier findings, while also extending their necessity and usefulness to detecting the nature and timing of ADRD disparities.

Our main analyses found that educational attainment was a reliable predictor of the onset of accelerated cognitive decline indicative of ADRD both before and after adjusting for demographic, behavioral, and medical risk factors common in the ADRD literature. However, while sex‐stratified analyses reported that inequalities were present in both males and females, the protective benefits of education were smaller in women. By addressing observational window bias, we found support for existing results that establish educational achievement as a factor in ADRD disparities, and we identified sizable educational inequalities in the onset of accelerated cognitive decline indicative of ADRD.

Here, we posit that educational attainment could have at least two main roles in the onset of accelerated cognitive decline and, thus, ADRD. First, education is believed to support healthy neural development and functional reorganization to improve lifelong cognitive reserve.^44^, ^45^ Educational attainment is also likely to predict the onset of other medical and psychiatric conditions, including depression and CVD, and indeed, here we found that lower educational attainment was associated with increased CVD risk, worse depressive symptoms, and higher risk of current and former smoking. As expected, after adjusting for these factors, the influence of education on the incidence of accelerated cognitive declines indicative of ADRD was reduced by approximately 25%. However, educational attainment also reflects and is supported by regional socioeconomic inequalities. These inequalities are also strongly associated with the provision of healthcare and public health services in the United States.

Minoritized race/ethnicity is generally found to be associated with increased risk of stress‐induced CVD,^46^ ADRD,^47^ and earlier mortality.^48^ One well‐known social mechanism for this process includes structural racism,^49^ resulting in reduced access to educational attainment, fewer chances to enter occupations with higher levels of control, reduced chances to live in healthy non‐polluted areas, and reduced ability to access healthcare services to reduce the impact of CVD. These factors could be important to consider in follow‐up studies. In addition, minoritized persons are often faced with interpersonal stressors consistent with overt racism, including, for example, increased risk of violence, discrimination, and stress due to racialization. These experiences are, in turn, believed to cause stress‐induced neuroinflammation.^50^ Interestingly, while Black participants were at the highest risk, risk was also higher for Hispanic participants and those reporting other races when compared to white participants. While these results are consistent with the view that difficulties faced by minoritized groups are partially due to differences in sociobehavioral and medical risk factors, they also suggest that these factors do not completely explain disparities.

In sex‐stratified analyses, we found that women from minoritized communities, including, notably, Black women, had higher overall risk of ADRD than white women; these differences were larger than the differences observed for comparisons of white and Black men. Some of this sex/gender disparity might emerge because men, on average, had higher overall risk of accelerated cognitive declines indicative of ADRD than women. However, results might also indicate that women from minority communities are susceptible to intersectional effects from multiple overlapping layers of stress, in accordance with intersectionality theory.^51^, ^52^ In this case, because stress and violence are potential causes of both racial and ethnic causes of ADRD, and because women are much more likely to be subjected to poverty, violence, and stress,^53^, ^54^ these results may simply reflect those factors. Biological mechanisms for intersectional results could include the potential for excess neuroinflammation relating to minority stress that overlaps with increased risk of tauopathy seen in women more generally, resulting in greater risk of tauopathic spread in this group. Regardless of the potential mechanisms underlying this association, this disparity deserves more careful attention in future work.

### 4.1 Strengths and limitations

The central strengths of this study include its multivariate approach and its novel focus on using longitudinal episodic memory data to examine the onset of accelerated cognitive decline in the used databases. Additional strengths of the study include its large sample size and its reliance on a novel method that proactively identifies patterns of cognitive decline consistent with ADRD, rather than relying on the presence of MCI or dementia, an approach that is sensitive to lengthy latency periods and suffers from high levels of misdiagnosis.

While this study has important strengths needed for studying social inequalities and longitudinal ADRD, several significant limitations might help contextualize the results from this study. First, while the survival analysis does account for right‐censoring, we do not account for study attrition due to the onset of ADRD. Studies have shown that the EM algorithms used in longitudinal models are robust to data missingness and more efficient than multiple imputation methods in longitudinal models and that individual random intercepts and slopes are effective indicators of cognitive performance.^55^ One problem apparent in prior studies is that estimates from random intercepts and slopes underpredict cognitive decline for individuals rated by proxy participants.^56^ One strength of a nested non‐linear approach is that data that are missing because people have accelerated rates of cognitive decline are, in accelerated models, captured by the parameter marking accelerated decline. As such, such models are less severely impacted by data that are missing as a result of accelerated rates of cognitive decline.

Second, while this model does identify accelerated rates of cognitive decline, it is unclear to what extent differential mortality in these groups influences results. If mortality is predominantly due to non‐cognitive factors that are also associated with educational attainment or ethnicity, then the current results will represent a conservative estimate of risk for accelerated cognitive decline. However, accelerated cognitive decline models cannot distinguish between accelerated declines because of ADRD or due to terminal decline. It remains unclear whether or not terminal cognitive decline is predominantly due to ADRD‐related processes. Since competing analysis is not yet readily integrated into these novel analytic methods, future work should seek to determine the sensitivity of these early markers of ADRD to competing risks including rates of death.

Third, while we could examine several behavioral factors, we did not integrate adjustments for several other factors, including, for example air‐pollution exposures, that have been identified as modifiable risk factors for ADRD.^57^

Fourth, we could not conduct an a priori power analysis because such power analyses require expectations for both expected incidence and power to detect the survival curve. As a result, we decided to use the largest and longest‐lasting study available to determine these risks despite also believing that, because ADRD is common enough, issues of statistical power should have been relatively minor. The current results therefore provide some of the data needed to complete power analyses in future studies.

Finally, while we assert that the symptoms shown here are indicative of declines expected in ADRD, we did not include the pathological data in this study necessary to validate that such accelerated cognitive declines are only evident in the presence of ADRD. Further work is warranted to determine the extent to which these accelerated cognitive declines are sensitive or specific to the presence of specific neuropathological biomarkers or whether they are also sensitive to other neurological conditions.

### 4.2 Conclusion

Livingston and colleagues^57^ suggested that 40% of the risk of ADRD onset, more than estimates of genetic predictors alone (estimated at roughly 23%^58^), was due predominantly to modifiable risk factors that were either indicators or correlates of educational attainment or race/ethnicity in the United States. Fundamental cause theory notes that social inequalities usually work through modifiable risk factors to influence health outcomes.^59^ As such, being able to identify social inequalities in the onset of ADRD is virtually required for any method seeking to show face validity. This study suggests that, after considering observational window bias, inequalities in ADRD‐related decline in episodic memory are evident and point in expected directions. Episodic memory decline is the main symptom seen in ADRD, and this study suggests that examination of rapid changes in episodic memory as the outcome can serve as a reliable basis for the study of ADRD.

## CONFLICT OF INTEREST STATEMENT

The authors declare no conflicts of interest. Author disclosures are available in the Supporting information.

## CONSENT STATEMENT

This study is a secondary analysis of deidentified data that are publicly accessible online (https://hrs.isr.umich.edu/about). The Institutional Review Board deemed that this study did not involve research on human subjects (CORIHS‐A#498619). Information on the HRS's consent process is available at: https://hrs.isr.umich.edu/publications/biblio/9048.

## Supporting information

Supporting Information

Supporting Information
