# Supplementary material for: Inequalities in accelerated cognitive decline: Resolving observational window bias using nested non‐linear regression
Source: Alzheimers Dement. 2024 Jul 12;20(8):5540–50. doi: 10.1002/alz.14053 (PMC11350020; doi:10.1002/alz.14053)
Supplement: Supplementary file 1 — Supporting Information [file ALZ-20-5540-s001.docx]

**Supplemental Appendix**

**Supplemental Quasi-Code 1:** Textual code showing the overall structure of the model estimated in the study

menl y = {int1:}+{surv1:} , define(int1: *…* U0[ID] c.*timescale*#U1[ID]) define(surv1: (({a00})*max(*b_t*-(exp({surv_c1: … }-{a11}**b_0*)),0))) cov(U0 U1, uns) stddev init({a00}=-1) [pw=Weight]

**Supplemental Figure 1.** Flow chart for sample selection, Health and Retirement Study

**Supplemental Table 1.** Differences between those who completed the full set of waves as compared to those who did not complete alongside multivariable analysis of the attrition rate, Health and Retirement Study

|  | Attritor |  | Did not yet attrite |  | aHR (95% C.I.) |
| --- | --- | --- | --- | --- | --- |
| Episodic Memory, words | 10.85 (3.06) |  | 9.54 (3.56) |  | 0.926 (0.921-0.932) |
| Age, years | 5.68 (4.80) |  | 15.36 (11.14) |  | 1.048 (1.046-1.051) |
| Educational Attainment |  |  |  |  |  |
| Less than high school | 11.6% |  | 25.7% |  | 1.000 |
| High school | 30.0% |  | 34.8% |  | 1.001 (0.956-1.047) |
| Some College | 28.4% |  | 21.7% |  | 1.055 (1.000-1.112) |
| University Degree | 30.0% |  | 17.8% |  | 1.039 (0.978-1.104) |
| Race/Ethnicity |  |  |  |  |  |
| White | 70.5% |  | 80.9% |  | 1.000 |
| Black | 12.6% |  | 10.1% |  | 0.858 (0.813-0.905) |
| Other | 6.3% |  | 2.9% |  | 0.989 (0.872-1.122) |
| Hispanic | 10.6% |  | 6.2% |  | 0.759 (0.706-0.817) |
| Male | 50.3% |  | 50.3% |  | 1.210 (1.149-1.274) |
| Wealth, ln($ + 1) | 8.00 (2.99) |  | 7.89 (2.94) |  | 0.970 (0.964-0.976) |
| Height, meters | 1.71 (0.10) |  | 1.70 (0.10) |  | 0.910 (0.709-1.168) |
| Cardiovascular Disease | 0.63 (0.77) |  | 0.89 (0.87) |  | 1.072 (1.053-1.091) |
| Stroke | 2.8% |  | 6.6% |  | 1.333 (1.274-1.394) |
| In the Labour Force | 77.5% |  | 42.9% |  | 0.832 (0.799-0.867) |
| Depression, Symptoms | 1.43 (1.98) |  | 1.53 (2.00) |  | 1.026 (1.016-1.035) |
| Smoking Status |  |  |  |  |  |
| Never Smoker | 46.0% |  | 38.3% |  | 1.000 |
| Former Smoker | 33.9% |  | 40.3% |  | 1.162 (1.118-1.207) |
| Current Smoker | 20.1% |  | 21.4% |  | 1.668 (1.576-1.766) |
| Alcohol intake, drinks/week | 1.35 (2.00) |  | 1.16 (2.09) |  | 0.966 (0.957-0.976) |

**Note:** Results are presented as mean (standard deviation) or using percentages (%). aHR: multivariable-adjusted hazards ratio; C.I.: confidence interval.

**Supplemental Table 2.** Characteristics of sample stratified by completeness of information on covariates.

| Sample Characteristics | Analytic Sample (n = 32441) |  | Incomplete Information (n = 911) |  | P |
| --- | --- | --- | --- | --- | --- |
| Episodic Memory, words | 10.3 (3.33) |  | 9.34 (3.59) |  | <0.001 |
| Age, years | 9.63 (9.32) |  | 9.3 (8.8) |  | <0.001 |
| Educational Attainment |  |  |  |  |  |
| Less than high school | 5643 (17.4%) |  | 324 (35.5%) |  | <0.001 |
| High school | 10350 (31.9%) |  | 245 (26.9%) |  |  |
| Some College | 8326 (25.7%) |  | 158 (17.3%) |  | <0.001 |
| University Degree | 8123 (25%) |  | 185 (20.3%) |  | <0.001 |
| Race/Ethnicity |  |  |  |  |  |
| White | 24199 (74.6%) |  | 422 (47.1%) |  | 0.23 |
| Black | 3764 (11.6%) |  | 93 (10.4%) |  | 0.11 |
| Other | 1605 (4.9%) |  | 51 (5.6%) |  | 0.87 |
| Hispanic | 2874 (8.9%) |  | 331 (36.9%) |  | <0.001 |
| Male | 16281 (50.2%) |  | 467 (51.3%) |  |  |
| Ln-Wealth | 7.95 (2.97) |  | 6.31 (3.94) |  | <0.001 |
| Height, meters | 1.7 (0.1) |  | 1.71 (0.1) |  | 0.28 |
| Cardiovascular Disease | 0.74 (0.82) |  | 0.74 (0.86) |  | 0.52 |
| Stroke | 1429 (4.4%) |  | 40 (4.4%) |  | 0.10 |
| In the Labour Force | 20580 (63.4%) |  | 274 (51.4%) |  | 0.04 |
| Depressive Symptoms | 1.48 (1.99) |  | 1.84 (2.25) |  | <0.001 |
| Smoking Status |  |  |  |  |  |
| Never Smoker | 13838 (43%) |  | 401 (57.2%) |  |  |
| Former Smoker | 11757 (36.6%) |  | 192 (27.4%) |  | <0.001 |
| Current Smoker | 6564 (20.4%) |  | 108 (15.4%) |  | <0.001 |
| Number of drinks per week | 1.28 (2.04) |  | 1.08 (1.98) |  | <0.001 |

**Note**: P-values test the difference between groups and are derived from non-parametric trend tests.

**Supplemental Table 3.** All model parameters estimated using nonlinear mixed effects modeling to examine onset of accelerated cognitive declines after a nodal onset

| **Survival Component** | **aNIR** | **95% C.I.** | **P** |
| --- | --- | --- | --- |
| Educational Attainment |  |  |  |
| Less than High School | 1.000 |  |  |
| High School Diploma | 0.148 | 0.104-0.21 | <0.001 |
| Some College | 0.112 | 0.079-0.16 | <0.001 |
| University Degree | 0.084 | 0.058-0.12 | <0.001 |
| Race/Ethnicity |  |  |  |
| White | 1.000 |  |  |
| Black | 3.090 | 2.793-3.418 | <0.001 |
| Other | 1.894 | 1.66-2.162 | <0.001 |
| Hispanic | 2.263 | 2.029-2.526 | <0.001 |
| Male | 1.656 | 1.6-1.714 | <0.001 |
|  |  |  |  |
| **Functional Correlates** | **Coef.** | **SE** | **P** |
| Unfamiliarity | -0.328 | 0.023 | <0.001 |
| Age, years | 0.058 | 0.003 | <0.001 |
| Mean Baseline Episodic Memory | 11.135 | 0.028 | <0.001 |
| Node Constant | 0.364 | 0.181 | 0.044 |
| Mean Rate of Accelerated Cognitive Decline | -0.797 | 0.003 | <0.001 |
|  |  |  |  |
| Random Intercepts | 2.284 | 0.013 |  |
| Random Slopes | 0.116 | 0.002 |  |
| Corr(Int., Slope) | -0.477 | 0.010 |  |
|  |  |  |  |
| Residual | 2.250 | 0.005 |  |

**Note**: Results from segmented regression models. Results here are consistent with those shown in Table 1, Demographics Model.
